# Nitric Oxide Synthase Activity Correlates with OGG1 in Ozone-Induced Lung Injury Animal Models

**DOI:** 10.3389/fphys.2017.00249

**Published:** 2017-04-27

**Authors:** Suqin Zhang, Jianhua Li, Yuqin Li, Yufeng Liu, Hongxiang Guo, Xiaoli Xu

**Affiliations:** ^1^Department of Pediatrics, First Affiliated Hospital of Zhengzhou UniversityZhengzhou, China; ^2^Department of General Surgery, First Affiliated Hospital of Zhengzhou UniversityZhengzhou, China

**Keywords:** 8-OxoG, OGG1, lung injury, NOS, arginase

## Abstract

**Background:** NO is an important cellular signaling molecule which is derived from L-arginine by nitric oxide synthase (NOS) and the effects of NOS signaling in lung injury is conflicting. The present study was designed to observe the effect of NOS and Arginase signaling in the occurrence and development of lung injury and its mechanism.

**Methods:** An ozone-stressed lung injury animal model was established by exposure to 2.0 ppm O_3_ for 30 min every day for consecutive 12 day with or without the administration of NO precursor L-arginine or non-selective NOS inhibitor N-nitro-L-arginine methyl ester (L-NAME). Then, the lung histopathology, the releases of inflammatory mediators and the production of ROS were assayed by immunohistochemistry, ELISA and flow cytometry respectively. The activities and expression of NOS and Arginase were assayed by biochemical methods and western blot. Correspondingly, the release of 8-oxoguanine glycosylase 1(8-OxoG) and 8-oxoguanine glycosylase 1 (OGG1) were assayed by ELISA and western blot. The correlation between NOS/Arginase signaling with 8-OxoG/ OGG1 was also analyzed by Pearson correlation coefficients and immunofluorescence in NOS deficient bronchial epithelial cells.

**Results:** In ozone-induced rat lung injury models, lung inflammation as well as lung architecture was disrupted in a time dependent manner. Ozone treatment with L-arginine showed a substantial attenuation of adverse lung histopathological changes and treatment with L-NAME promoted the inflammation and remodeling. Importantly, the expression of NOS was promoted by L-arginine and inhibited by L-NAME and the expression of Arginase was promoted by L-NAME treatment. Further, we observed significantly higher levels of 8-OxoG and lower levels of OGG1 in ozone group which was reversed by L-arginine and promoted by L-NAME. The expression of NOS is closely related with 8-OxoG /OCG1.

**Conclusion:** These findings give further evidence that the NOS signaling is related with base excise repair.

## Introduction

Our body communicates with external environment mainly through respiratory tract, gastrointestinal tract, and skin. Among them lungs are more vulnerable to injury induced by inhaled toxicants on account of its large surface that is constantly exposed to the external environment. Atmospheric dust, CO, SO_2_, O_3_, heavy metals, and bacteria, viruses, fungi can easily enter the body through respiratory tract and induce airway stress damage (Wang et al., 2015). Oxidative stress and ROS induced by inflammation can modify proteins, lipids, and DNA. High level of oxidative stress lead to severe damage to DNA and eventually lead to cellular senescence or death. Among these 4 bases of DNA: Cytosine(C), Thymine(T), Adenine(A), and Guanine(G), oxidative damage mostly occurs in guanine base due to its low redox potential. 8-oxoguanine (8-oxoG) is one of the most common DNA modifications caused by ROS which can lead to G to T and C to A substitutions in the genome. The 8-oxoG level in serum and sputum is one of the important biomarker for advanced asthma and COPD (Deslee et al., 2010; Yang et al., 2014). 8-oxoguanine glycosylase 1 (OGG1) catalyzes the first step in the base excision repair pathway (BER), responsible for detecting and excising the mutagenic base product with 8-oxoG, when 8-oxoG is formed in guanine-rich sequences (Belanger et al., 2016; Fleming et al., 2017).

Nitric oxide (NO) is a reactive free radical which acts as an important regulator and biological mediator of numerous processes in the nervous, immune, and cardiovascular systems and the numerous roles of NO in respiratory pathophysiology have been extensively reviewed (Girotti et al., 2016; Zhang et al., 2016). NO has potential therapeutic uses in viral exacerbations of asthma and COPD, since it inhibits and suppresses virus replication and production of several cytokines and chemokines in airway epithelium (Tsicopoulos et al., 2013). What's more, NO strongly inhibits the contractions of airway and blood vessels (Hasaneen et al., 2003). NO is formed from L-arginine by nitric oxide synthase (NOS). Arginine is a semi-essential amino acid which serves as an essential precursor for protein, polyamines, creatinine urea, ornithine, proline, glutamate, and NO biosynthesis. Arginine plays remarkable role in cellular metabolism and regulation (Rath et al., 2014). The discovery that L-arginine is the essential and important precursor for NO biosynthesis precipitated research into the role of arginine in various health conditions including normal physiological and pathological conditions (Durante et al., 2007; Kozan et al., 2016).

Both NOS and Arginase serve as competitive inhibitor for each other for substrate arginine so increasing activity of arginase decreases arginine substrate for NOS and reduced NO production (Bratt et al., 2011). And new treatments of pulmonary hypertension in hemolytic disorders is aimed at improving arginine and NO bioavailability through arginase inhibition (Hsu et al., 2007). In the allergic airways disease, the arginase activity is inhibited and that may cause NO homeostasis imbalance (Ogino et al., 2013). All the proofs above suggest the protective role of NOS/NO in airway homeostasis. However, the data about the protective effects of NO in acute lung injury are somewhat conflicting, that is, NO may relate to the development of acute lung injury by NOS activity (Gross et al., 2015).

To probe the role of NOS in lung injury, in the context, we observed the expression characteristics of NOS and Arginase in ozone-induced lung injury animal models after the administration of L-arginine, as a NO precursor and N-nitro-L-arginine methyl ester (L-NAME), as a NOS inhibitor. Ozone stress is the commonly used factor to induce oxidative stress and subsequent DNA damage in rodent lungs (Rumsey et al., 2016). The relationship of NOS and Arginase with 8-oxoG/OGG1 accumulation was also examined.

## Methods and materials

### Ethics statement

This study was accepted by the Animal Care and Committee of Zhengzhou University. Moreover, this study was conducted in compliance with the guidelines of the National Institutes of Health for the care and the use of laboratory animals. All animals in the study groups survived throughout the experimental period.

### Animals and experimental design

Sprague Dawley (SD) rats (180–220 g) were purchased from Zhengzhou University. Animals were divided into four groups including control group, ozone group, control+ L-arginine group and ozone + L-NAME group and there were 20 animals or so in each group. The animals in ozone group were exposed to 2.0 ppm O_3_ for 30 min and room air with a natural light cycle for consecutive 12 days and the control group was given the same treatment without ozone stress. Animals in L-arginine group were intravenously injected 60 mg/kg of L-arginine and L-NAME group were intravenously injected 15 mg/kg of L-NAME for consecutive 12 days respectively at the first day after ozone stress while animals in control group and ozone group were injected saline for consecutive 12 days as a place. The animals (4 rats) from different group were observed at different time points (Day 4, Day 8, and Day 12).

### Immunohistochemistry and immunofluorescence

To evaluate the severity of lung injury, the right lobe lungs were obtained at Day 4, 8, 12 respectively. Lungs were filled with air and removed from the animal model. Later on it was fixed in 4% paraformaldehyde, embedded in paraffin and cut into 5 μm sections. Lung sections were immunostained with a polyclonal rabbit anti α-smooth muscle actin (α-SMA) antibody at a dilution of 1:200. As a smooth muscle marker, a polyclonal rabbit anti NOS antibody at a dilution of 1:200 and a polyclonal rabbit anti OGG1 antibody (Santa Cruz Biotech, USA) at a dilution of 1:200 at 37°C for 2 h. The slides were washed and incubated with biotinylated goat anti rabbit IgG, Cy3-labeled or FIFC-labeled lgG (Boster, Wuhan, China) for 1 h and washed again. After washing in PBS, the signal was detected with 3, 3′-diaminobenzidine (Dingguo, Beijing, China) or fluorescence microscope.

### Enzyme-linked immunosorbent assay (ELISA)

Rats were injected with an intraperitoneal injection of sodium pentobarbitone (100mg/kg). The BALF was obtained by washing the airway with 1mL of PBS into the lungs. The recovery is 80%. ELISA were performed on BALF using kits for rat-specific TNFα, TGFβ1, IL10 (Boster, Wuhan, China) and MPO ELISA Kits (CUSABIO, MD, USA), according to the manufacturer's instructions. Lung tissues were than homogenized and centrifuged at 10,000 g for 5 min and the level of OGG1 was determined using ELISA provided by CUSABIO (USA). The DNA was extracted from lung tissues and the amount of 8-hydroxy-2-deoxyguanosine (8-ohdG) was determined using ELISA.

### Measurement of ROS

DCFH-DA (Dichloro-dihydro-fluorescein diacetate) is a quantitative method for oxidative stress assessment. Its Probes was used to measure the ROS according to the manufacturer's protocol. The lung tissue after the proper treatment were incubated in pre-warmed PBS containing the probes in a final working concentration of 5 μM for 30 min at 37°C. Flow cytometry detected the ROS fluorescence intensity (488/518 nm).

### Determination of arginase and NOS activities

Arginase is a manganese containing enzyme. It converts L-arginine into urea and L-ornithine. The supernatant of lung tissue homogenate was reacted with reaction solution (containing L-arginine) to detect the product of reaction, L-ornithine and the absorbance was measured at 570 nm according to an Arginase Activity Assay kit (BioVision, CA, USA). The final products of nitrates from NOS reaction with L-arginine, represented the activity of NOS, was measured at 540 nm according to a NOS Activity Assay kit (Novus Biologicals, CO, USA). The concentrations were determined by standard curves.

### Western immunoblotting

After protein concentrations of lung tissue homogenate were determined with a coomassie plus protein assay kit (Dingguo, Beijing, China). Then, 75 μg of proteins were separated by gel electrophoresis and then transferred onto PVDF membrane. Then membranes were blocked with 5% skim milk and incubated with rabbit polyclonal anti-Arginase I, anti-NOS and anti-OGG1. Later on membranes were washed and incubated with corresponding secondary IgG for 1 h at room temperature. Lastly, antibody–antigen complexes were detected by using ECL chemiluminescent detection system. β-actin was used as a loading control. A densitometry analysis was performed by AlphaEase software (version: 2200).

#### NOS knockdown

siRNA oligonucleotides for NOS and control siRNA were purchased from abnova (Taiwan, China). Human bronchial epithelial cells were seeded on plates. Ninety percent confluent monolayer cultures were incubated with final concentration of 10 nM NOS siRNA or control siRNA using HiPerFect Transfection Reagent (QIAGEN, China) for 24 h.

### Statistical analysis

The data are presented by mean ± S.E. The statistical analyses were performed by using ANOVA for comparison between same group and the multiple treatment groups. The relationships between variables were explored by simple correlation and multiple regression analyses. *P* < 0.05 was considered as statistically significant.

## Results

### Lung inflammation and structural changes during lung injury

**Figure 1:**
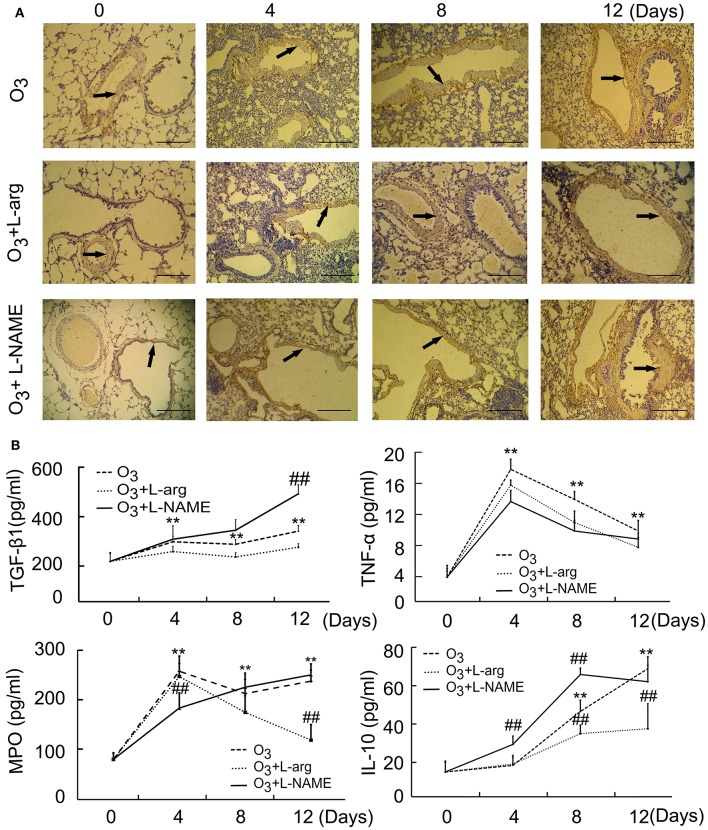
**The lung injury induced by ozone and treated with L-arginine and L-NAME was assayed by immunohistochemistry (A**, scale bar = 100 μM) and ELISA (*n* = 5, **B**). All groups were observed at Day 0, Day 4, Day 8, and Day 12 and 4 rats were observed at different time points. ^**^P < 0.01 vs. Day 0 of ozone group; ^##^*P* < 0.01 vs. the same time point of ozone group. Arrows represented α-SMA positive staining.

The results of microscopic immunohistochemistry staining images of lung sections showed that the bronchial and alveolar wall are complete, no inflammatory cell infiltration, and luminal stenosis were observed in normal control group (at Day 0, Figure 1A). However, in ozone stress group, infiltration of inflammatory cells in bronchial lumen, alveolar cavity and pulmonary interstitial were obviously increased. And some bronchial epithelial cells were found to fall off and the expression of smooth muscle biomarker was found to increase with time (Figure 1A). L-arginine had obvious protective effects on airway inflammation and structure at different time points and L-NAME obviously promoted the remodeling (Figure 1A).

In order to probe the possible molecular signal mechanism of NOS signaling, several major representative regulatory mediators involved in airway injury and remodeling were measured using ELISA. The results showed that the secretion of TGF-β 1 was enhanced from Day 4 after ozone stress and kept augmented during ozone stress and L-NAME promoted the production of TGF-β 1 at Day 12 after ozone stress when compared with ozone group (Figure 1B); the secretion of TNFα increased rapidly to peak at Day 4 and gradually decreased from peak after ozone stress, and L-arginine and L-NAME had no influence on the secretion of TNFα (Figure 1B); the activities of MPO had the same tendency with TNFα after ozone stress and L-NAME inhibited the activities of MPO at Day 4 and L-arginine inhibited the activities of MPO at Day 12 after ozone stress (Figure 1B); the secretion of IL-10 increased gradually from Day 8 after ozone stress and L-arginine inhibited the production of IL-10 at Day 8 and Day 12, while L-NAME promoted the production of IL-10 at Day 4 and Day 8 (Figure 1B).

**Figure 2:**
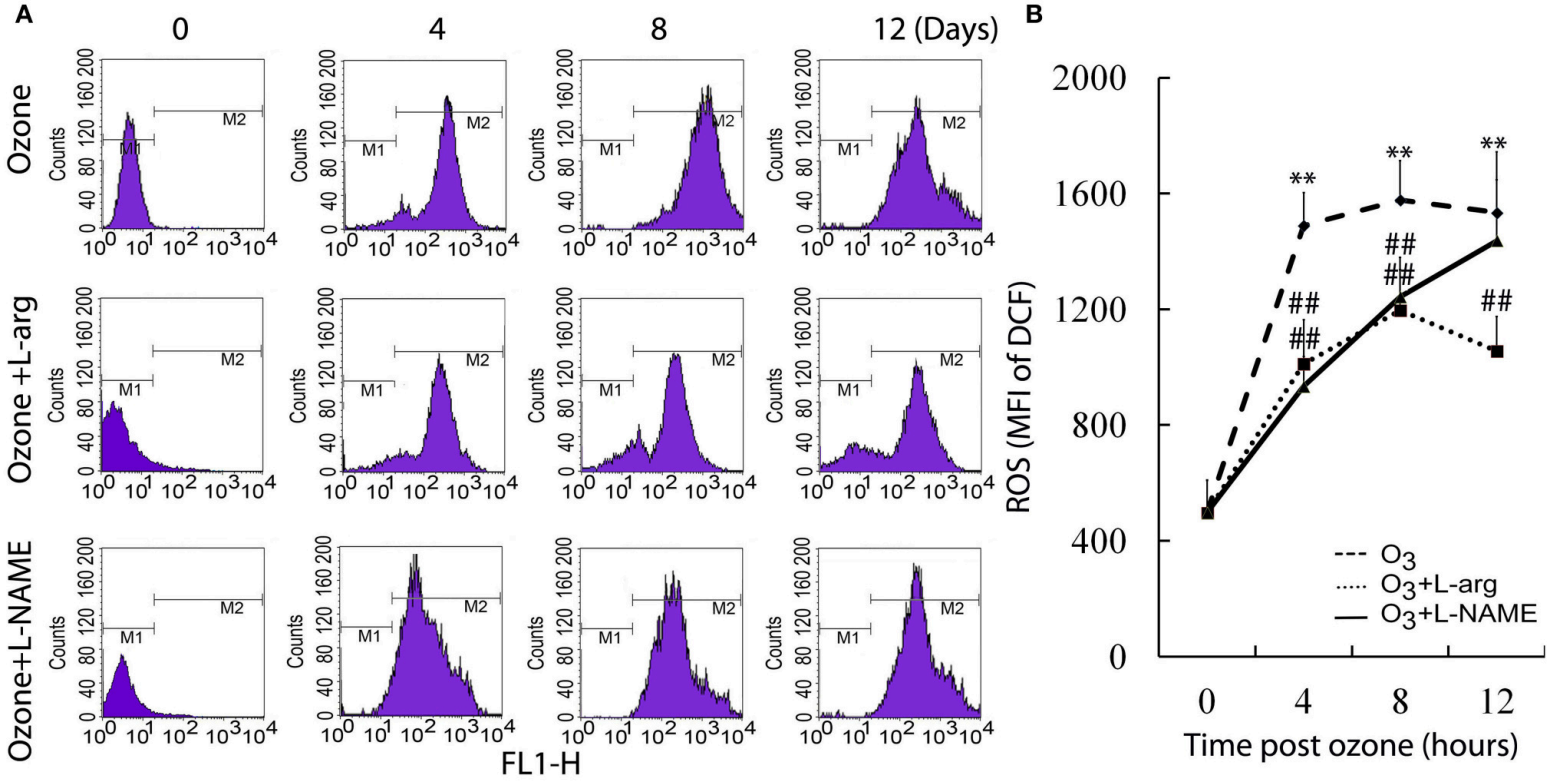
**The production of ROS was assayed by flow cytometry (***n*** = 4). (A)**, representative images. **(B)**, the average MFI. All groups were observed at Day 0, Day 4, Day 8, and Day 12 and 4 rats were observed at different time points. ^**^P < 0.01 vs. Day 0 of ozone group; ^##^*P* < 0.01 vs. the same time point of ozone group.

The ROS assay showed that ozone markedly enhanced the ROS production at different time points as determined using flow cytometry analysis (Figure 2A). Both L-arginine and L-NAME seem to inhibit the production of ROS (Figure 2B).

### NOS and arginase signaling during lung injury

**Figure 3:**
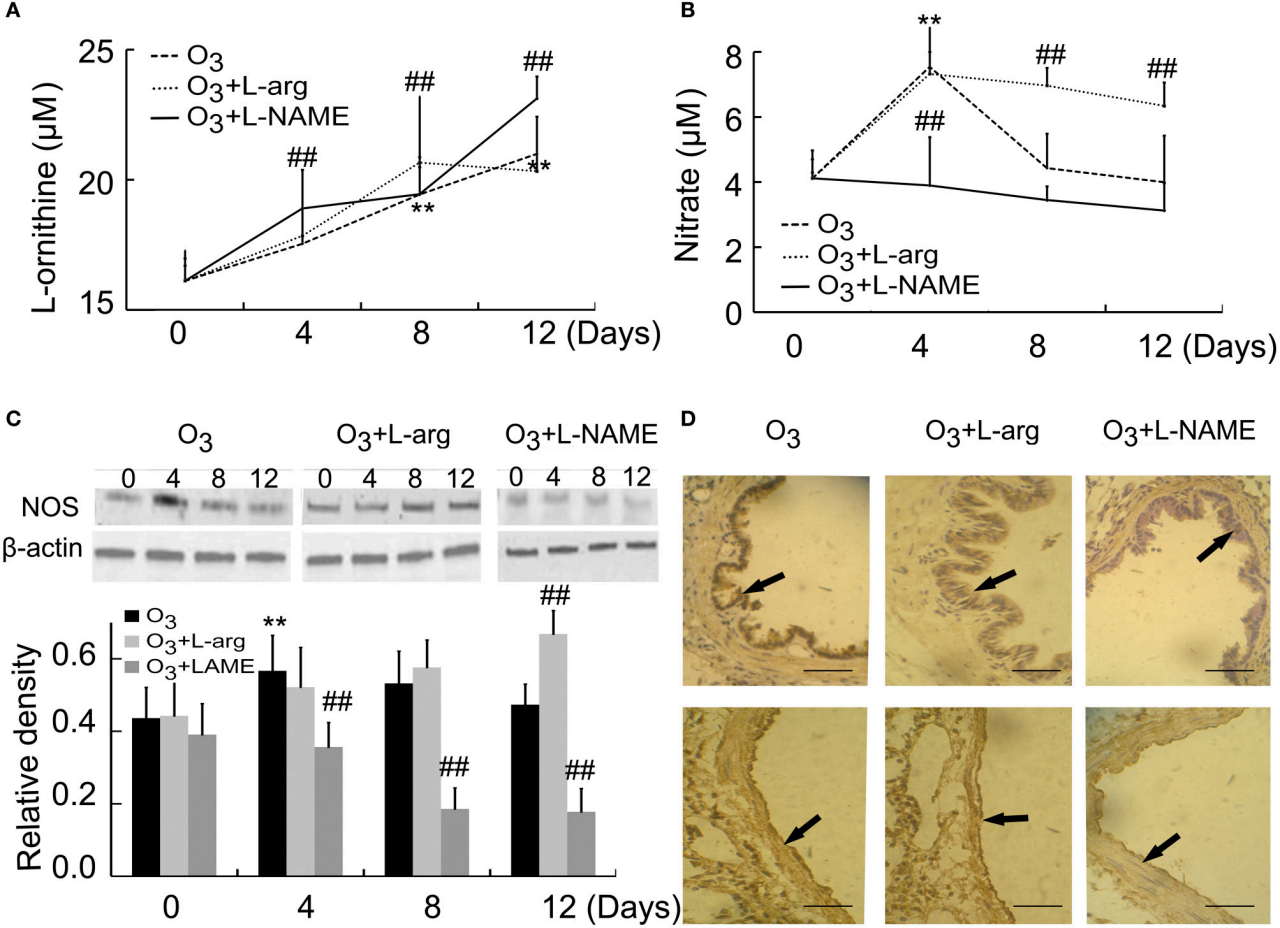
**The activities of Arginase (A)** and NOS **(B)** were assayed by detecting the concentrations of reaction products (*n* = 5) and the expression and distribution of NOS were assayed by Western blot (**C**, *n* = 4) and immunohistochemistry (**D**, scale bar = 50 μM). All groups were observed at Day 0, Day 4, Day 8, and Day 12 and 4 rats were observed at different time points. ^**^P < 0.01 vs. Day 0 of ozone group; ^##^*P* < 0.01 vs. ozone group. The distribution of NOS was observed at Day 12. Arrows represented NOS positive staining.

By using ELISA assay, our results showed that the contents of arginase increased with time (Day 8 and Day 12) in ozone group. L-arginine promoted the production of arginase at Day 8, but L-NAME promoted the production of Arginase at Day 4 and Day 12 after ozone stress (Figure 3A). On the other hand, the contents of NOS increased to the peak at Day 4 and decreased to the normal level from Day 8 in the ozone group. L-arginine promoted the production of NOS at Day 8 and Day 12, but L-NAME inhibited the production of NOS at Day 4 (Figure 3B). These *in vivo* data indicated that L-arginine promoted the ozone-inhibited NOS activity and L-NAME inhibited the production of NOS.

NOS were also assayed by western blot. Our data indicated that NOS levels initially increased at Day 4 after ozone stress and gradually decreased to normal level, but after treatment with L-arginine, NOS levels increased with time and after treatment with L-NAME, the production of NOS was inhibited from Day 4 when compared with the same time point of ozone group (Figure 3C). NOS was mainly distributed in epithelial cells and endothelial cells (Figure 3D).

### Production of 8-oxoG/OGG1 during lung injury

**Figure 4:**
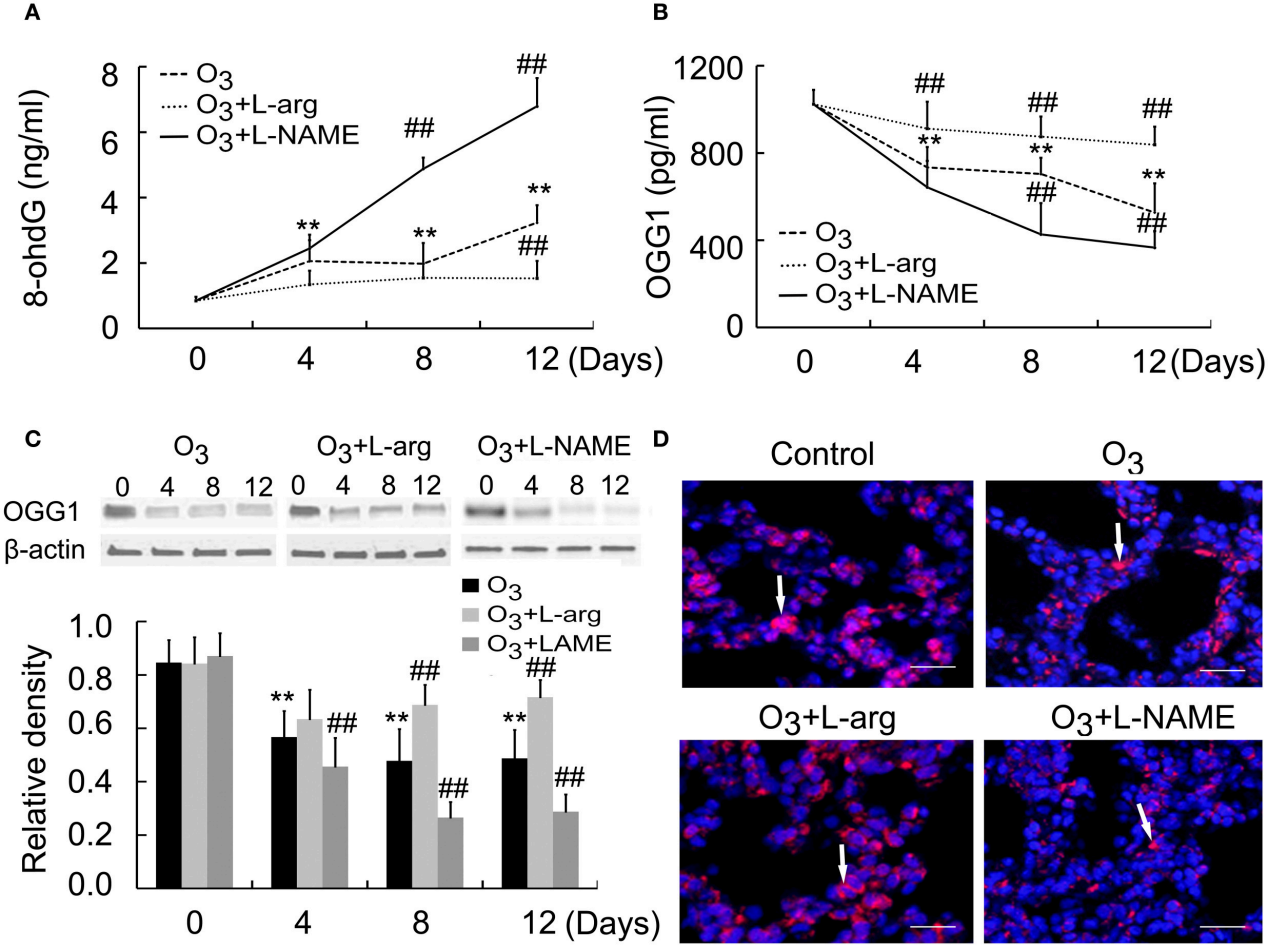
**The production of 8-ohdG (A)** and OGG1 **(B)** was assayed by ELISA (*n* = 5) and the protein expression and distribution of OGG1 were assayed by Western blot (**C**, *n* = 4) and immunofluorences (**D**, scale bar = 50 μM). All groups were observed at Day 0, Day 4, Day 8, and Day 12 and 4 rats were observed at different time points. ^**^P < 0.01 vs. Day 0 of ozone group; ^##^*P* < 0.01 vs. ozone group. The distribution of OGG1 was observed at Day 12. Arrows represented OGG1 positive staining.

8-OxoG is the most common DNA base lesions induced by ROS; so it is most commonly used as an indicator for oxidative stress (Sassa et al., 2016). With the help of ELISA assay, the 8-oxoG levels in DNA was evaluated in which specific antibodies against 8-ohdG (a nucleoside of 8-oxoG) was used. The 8-ohdg Levels were markedly increased in ozone group than control group at Day 0; But, L-arginine reduced the levels of 8-ohdG at Day 12, while L-NAME promoted the production of 8-ohdG at Day 8 and Day 12 after ozone stress (Figure 4A).

OGG1 is a constituent of the base excision repair (BER) system of DNA bases which primarily excizes the 8-oxoG base. The activities of the OGG1 were determined by using ELISA. The OGG1 level were lower in ozone group compared to control group at Day 0; But, L-arginine promoted and restored the levels of OGG1 from Day 4 and L-NAME inhibited the production of OGG1 at Day 8 and Day 12 after ozone stress (Figure 4B). Moreover, lung tissues were subjected to western blotting using anti-OGG1 antibodies. A 38 kDa protein corresponding to OGG1 were detected and decreased OGG1 were detected in ozone-induced lung injury animal models. L-arginine treatment promoted and restored the production of OGG1 at Day 8 and Day 12 and L-NAME inhibited the production of OGG1 from Day 4 after ozone stress (Figure 4C). By using immunofluorescence, we found OGG1 obviously decreased in ozone-stressed lung injury models, which was reversed by administration of L-arginine and aggravated by administration of L-NAME. Besides, OGG1 was mainly localized in epithelial cells and endothelial cells and exhibited cytoplasmic immunoreactivities (Figure 4D).

### The NOS signaling is related to OGG1/8-oxoG

**Figure 5:**
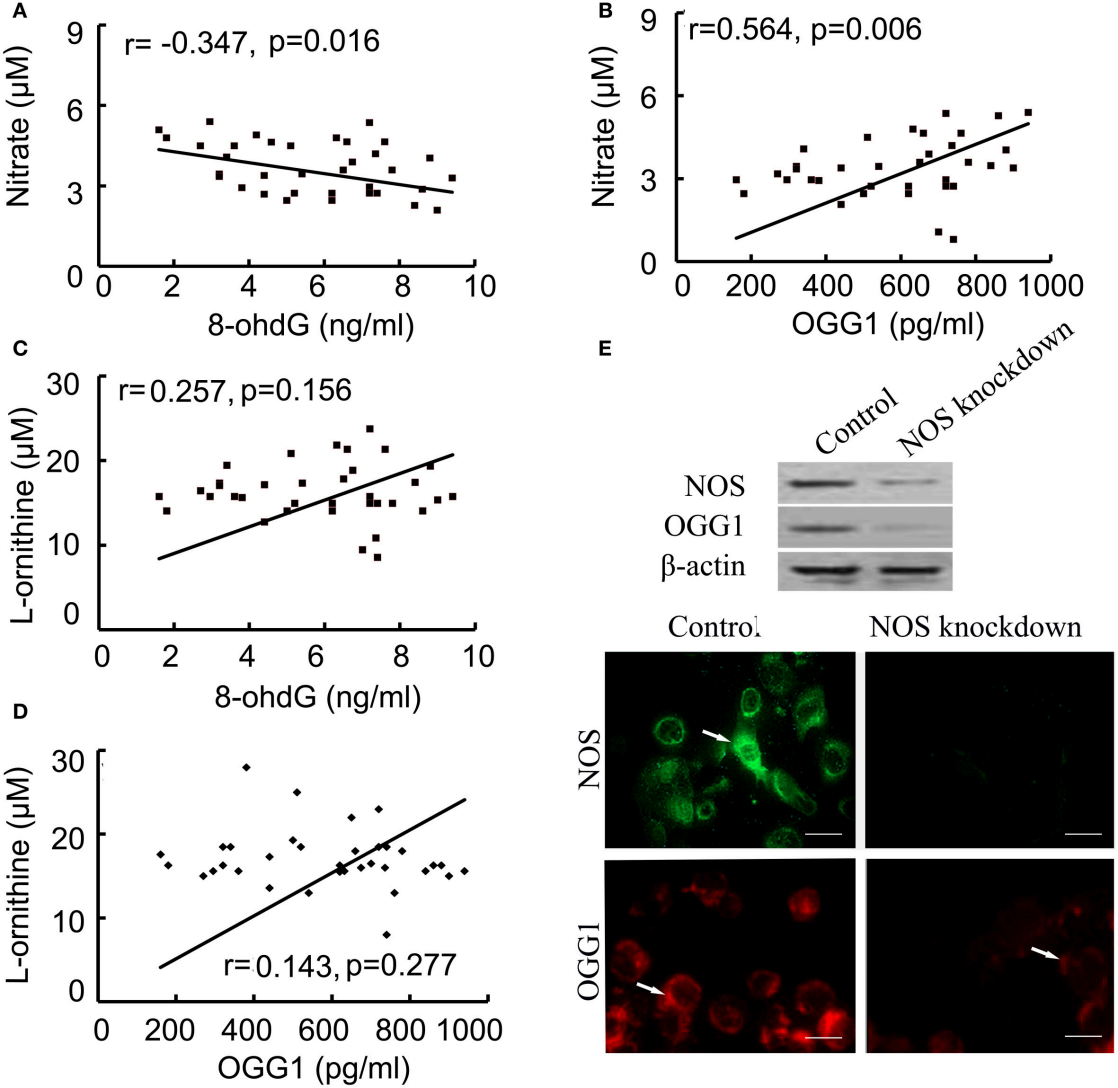
**Correlation between NOS and 8-ohdG/OGG1 was analyzed by Pearson correlation coefficients and immunofluorences. (A,B)**: correlation between NOS and 8-ohdG/OGG1; **(C,D)**: correlation between Arginase and 8-ohdG/OGG1; **(E)**: correlation between NOS and OGG1 in NOS-deficient cells (scale bar = 50 μM). The results showed that NOS was closely associated with OGG1. Arrows represented NOS (green) and OGG1 (red) positive staining respectively.

Correlated analysis results showed NOS was positively associated with OGG1, while negatively correlated to 8-ohdG contents. In multivariate linear model, the positive relationship between NOS and OGG1 level was persistent during injury process (Figures 5A,B). On the other hand, we found that there was no correlation between the expression of arginase and 8-ohdG/OGG1 (Figures 5C,D). These data indicated that NOS signaling was closely related with 8-ohdG/OGG1.

To further investigate the association between NOS and OGG1, NOS siRNA or negative control was transfected into human bronchial epithelial cells. After confirming the successful downregulation of NOS in cells by using western blot and indirect immunofluorescence (IFA), the expression of OGG1 was examined by western blot and IFA (Figure 5E). The results showed that OGG1 expression was down-regulated by NOS knockdown, suggesting that NOS signaling was closely related with OGG1.

## Discussion

Ozone-stress induced lung injury models were commonly used models in acute and chronic lung injuries. Usually, ozone stress during 4 days is recognized as acute lung injury and prolonged stress will induce chronic lung injury (Xiang et al., 2014). It is well known that NO can play a role in the development of acute lung injury by ROS activity (Li et al., 2015). The results of the present study showed that ozone stress led to infiltration of inflammatory cells and tracheal remodeling with stress time. L-arginine had obvious protective effects on airway structure and inflammation and L-NAME obviously promoted the inflammation and remodeling.

Proinflammatory cytokines TGF-β1 after lung injury contributes in the formation of pulmonary fibrosis through receptor mediated phosphorylation of Smad2 and Smad3 (Wang et al., 2016). The results of the present study showed that TGF-β 1 was enhanced from Day 4 after ozone stress and kept augmented during ozone stress and L-NAME promoted the production of TGF-β 1 at Day 12 induced by ozone, at the same time, L-NAME promoted the production of arginase at Day 12, indicating that arginase was associated with airway remodeling.

TNF-α is an important proinflammatory cytokine to enhance the transcription of other proinflammatory cytokines through PI3K/Akt signaling pathway and then through a chain of inflammatory reaction result into persistence of inflammation in lung (Park et al., 2014). Although the secretion of TNFα increased rapidly and kept high levels during ozone stress, L-arginine, and L-NAME had no influence on the secretion of TNFα.

Myeloperoxidase (MPO) is a main component of azurophilic granules of neutrophil. MPO are used by the neutrophil to kill bacteria and other pathogens so measuring MPO directly indicates neutrophil presence whereas indirectly indicates lungs injury (Hoenderdos et al., 2016). The results of the present study showed L-NAME inhibited the activities of MPO at acute phase after ozone stress, and L-arginine inhibited the activities of MPO at chronic phase, indicating NO inhibition and activation at different phase may alleviate lung injury.

The potent anti-inflammatory and immunosuppressive cytokine IL-10, helps control inflammation and modulate adaptive immune responses following tissue damage in numerous inflammatory diseases (Hesse et al., 2004). The present study results showed that IL-10 secretion increased rapidly with time after ozone stress. L-arginine inhibited the production of IL-10, while L-NAME promoted the production of IL-10, indicating that NOS signaling promoted inflammation.

The presence of pro-inflammatory cytokines stimulates the NOS to synthesizes large amount of NO, which may contribute to severe lung injury. A report showed that NOS plays a proinflammatory role in acute hyperoxic lung injury by using NOS deficient mice (Shouval et al., 2014). The present study showed NOS correlates with rat inflammation at acute phase after ozone stress, probably through its proinflammatory role.

As for the relationship between NOS and Arginase, the present study results show a marked increase and peak of NOS at day 4 after ozone stress, followed by steady decline to normal level after ozone stress from Day 8. L-arginine promoted the production of NOS, while L-NAME inhibited the production of NOS. However, according to the observation of Arginase, L-NAME obviously promoted the production of Arginase at Day 4 and Day 12 and inhibited the production of Arginase at Day 8. The obstruction and hyperresponsiveness of the airways increased with enhanced activity of arginase by decreasing in the synthesis of bronchodilatory NO that caused by its competition with NOS for their common substrate (Maarsingh et al., 2009); indicating NOS signaling may influence the production of Arginase via different pathways.

Substantial evidence has accumulated to show that oxidative damage occurs as a consequence of exposure to polluted environment (Bai et al., 2016). The results from the present study showed that 8-oxoG/OGG1 activities were associated with NOS activity. However, the mechanism remains unclear. A report showed that the reduction of NO level and NOS expression prevented (ADP-Ribose) Polymerase 1 (PARP-1) activation (Martínez-Romero et al., 2012). PARP-1 is the key factor to induce the activation of BER (Dziaman et al., 2014). There is still some limitations in current study. First, although we revealed the relationship between NOS signaling and BER, further detailed studies including NO and L-arginine production and the mechanism may be necessary. Second, *in vitro* NOS deficient studies should be carried out to verify the association between the NOS signaling and BER.

In summary, it is well established that the both arginase and NOS enzymes compete for their common substrate arginine. The enhanced activity of arginase is not only lead to impair NO production but also it contributes to the enhanced production of reactive oxygen species by NOS. This is the 1st study showing that NOS pathway is being involved in the BER in the lung.

## Author contributions

Conception and design: JL; Collection and assembly of data: SZ, YLi, YLiu; Data analysis and interpretation: HG, XX; Manuscript writing: JL; Final approval of manuscript: All authors.

## Funding

The project was funded by Foundation of cutting-edge technologies of Henan province (162300410114).

### Conflict of interest statement

The authors declare that the research was conducted in the absence of any commercial or financial relationships that could be construed as a potential conflict of interest.
